# Brain volume reductions in adolescent heavy drinkers

**DOI:** 10.1016/j.dcn.2014.02.005

**Published:** 2014-02-22

**Authors:** Lindsay M. Squeglia, Daniel A. Rinker, Hauke Bartsch, Norma Castro, Yoonho Chung, Anders M. Dale, Terry L. Jernigan, Susan F. Tapert

**Affiliations:** a VA San Diego Healthcare System, La Jolla, CA, USA; b University of California, San Diego, Department of Psychiatry, La Jolla, CA, USA; c University of Southern California, Imaging Genetics Center, Institute for Neuroimaging and Informatics, Los Angeles, CA, USA; d Yale University, Department of Psychology, New Haven, CT, USA; e University of California, San Diego, Department of Radiology, La Jolla, CA, USA; f University of California, San Diego, Department of Cognitive Science, La Jolla, CA, USA; g University of California, San Diego, Center for Human Development, La Jolla, CA, USA

**Keywords:** Adolescence, Alcohol abuse, Brain development, Neuroimaging, Magnetic resonance imaging, QUARC

## Abstract

**Background:**

Brain abnormalities in adolescent heavy drinkers may result from alcohol exposure, or stem from pre-existing neural features.

**Methods:**

This longitudinal morphometric study investigated 40 healthy adolescents, ages 12–17 at study entry, half of whom (*n* = 20) initiated heavy drinking over the 3-year follow-up. Both assessments included high-resolution magnetic resonance imaging. FreeSurfer was used to segment brain volumes, which were measured longitudinally using the newly developed quantitative anatomic regional change analysis (QUARC) tool.

**Results:**

At baseline, participants who later transitioned into heavy drinking showed smaller left cingulate, pars triangularis, and rostral anterior cingulate volume, and less right cerebellar white matter volumes (*p* < .05), compared to continuous non-using teens. Over time, participants who initiated heavy drinking showed significantly greater volume reduction in the left ventral diencephalon, left inferior and middle temporal gyrus, and left caudate and brain stem, compared to substance-naïve youth (*p* < .05).

**Conclusion:**

Findings suggest pre-existing volume differences in frontal brain regions in future drinkers and greater brain volume reduction in subcortical and temporal regions after alcohol use was initiated. This is consistent with literature showing pre-existing cognitive deficits on tasks recruited by frontal regions, as well as post-drinking consequences on brain regions involved in language and spatial tasks.

## Highlights

• Pre-existing frontal brain volume differences were found in future drinkers.
• Adolescent drinkers showed greater brain volume reduction post-alcohol initiation.
• Volume reduction occurred in subcortical and temporal regions.
• QUARC is a useful tool for quantifying longitudinal brain volume changes.

## 1 Introduction

Alcohol use is exceedingly common during adolescence, with rates of past year alcohol use in the US increasing from 24% to 64%, and past year drunkenness rising from 9% to 45% from ages 12 to 18 (Johnston et al., 2013). Furthermore, almost a quarter of US 18 year olds report heavy episodic drinking, defined as consuming five or more drinks on one occasion, during the past two weeks (Johnston et al., 2013). These high rates of heavy alcohol use are concerning, as the adolescent brain undergoes extensive morphometric and functional maturation, including decreases in gray matter and increases in white matter volume (Giedd, 2004, Giedd et al., 1999, Gogtay et al., 2004, Luna and Sweeney, 2004, Spear, 2000). Gray matter reductions (i.e., cortical thinning) begin during early adolescence (approximately ages 12–14) and are generally considered to be related to pruning of excess neurons, changes in the extracellular matrix, and white matter encroachment (Paus, 2005), beginning primarily in posterior brain regions and progressing to more anterior regions (Gogtay et al., 2004) with decreases in dorsal prefrontal cortical volume continuing into early adulthood (mid-20s) (Sowell et al., 2001). In tandem with cortical thinning, white matter volume increases over adolescence, due to myelination of white matter tracts (Barnea-Goraly et al., 2005, Giedd et al., 1999, Pfefferbaum et al., 1994). These co-occurring processes are an integral component of neurocognitive development, creating more localized and efficient information processing and improved cognition (Squeglia et al., 2013). Because of these extensive maturational changes, the developing adolescent brain may be more vulnerable to the deleterious effects of alcohol (Jacobus and Tapert, 2013).

Heavy alcohol use during adolescence has been cross-sectionally associated with disadvantages on several neuropsychological domains, including memory, executive functioning, visuospatial skills, and sustained attention (Brown et al., 2000, Giancola et al., 2001, Sher et al., 1997). Importantly, longitudinal studies have suggested an adverse influence of adolescent heavy drinking (initiated around ages 15–16) on the development of visuospatial processing, attention, and working memory (Hanson et al., 2011, Squeglia et al., 2009, Tapert et al., 2002). Furthermore, deficits on tasks of inhibitory functioning in substance-naïve youth have been related to initiation of heavy alcohol use by ages 17–18 (Squeglia et al., 2014), suggesting cognitive functioning is both predictive of, and affected by, alcohol use.

The underlying mechanism of these behavioral changes may be related to morphometric anomalies in brain volume or cortical thickness. Research using structural magnetic resonance imaging (MRI) has shown smaller hippocampal (De Bellis et al., 2000, Nagel et al., 2005), prefrontal cortex (De Bellis et al., 2005, Medina et al., 2008), and cerebellum (De Bellis et al., 2005, Lisdahl et al., 2013) volumes in heavy-drinking teens compared to non-using controls. In a recent longitudinal study in youth characterized before (age ∼17) and after (age ∼19) initiating heavy alcohol use, adolescents who began heavy drinking over the follow-up period showed accelerated cortical thinning of right middle frontal gyrus, as well as decreased white matter volume, when compared to demographically matched non-using teens (Luciana et al., 2013). No differences were found between groups before initiation, suggesting alcohol use was related to aberrant cortical thinning, as opposed to cortical thickness being predictive of initiation of alcohol use. Furthermore, widespread cortical thinning and volume reduction has also been reported in alcohol dependent adults in frontal, temporal, and occipital regions (Fortier et al., 2011, Pfefferbaum et al., 1997).

The goals of this study were to use a set of novel analytic approaches to carefully examine within-subjects changes in morphometry and quantify cortical volume changes over time in youth who remained non-drinkers compared to those who initiated heavy drinking. We hypothesized that adolescents who transitioned into moderate to heavy drinking would show smaller cortical volumes, similar as has been seen in adolescent drinkers (Luciana et al., 2013) and adult alcoholics (Fortier et al., 2011, Pfefferbaum et al., 1997).

## 2 Methods

### 2.1 Participants

The sample was obtained from a larger ongoing neuroimaging study of 296 adolescents examining neurocognition in youth at-risk for substance use disorders (Bava et al., 2010, Squeglia et al., 2012a, Squeglia et al., 2011, Wetherill et al., 2013a). Participants were recruited through flyers sent to households of students attending local middle schools, describing the study as a project looking at adolescent brain development in youth who do or do not use alcohol, and included major eligibility criteria, financial compensation ($170 for youth, $20 for parents), and contact information. Informed consent and assent were obtained, and included approval for youth and parents be contacted for follow-up interviews and scans. Eligibility criteria, substance use history, family history of substance use, developmental, and mental health functioning data were obtained from the youth, their biological parent, and one other parent or close relative. The study protocol was executed in accordance with the standards approved by the University of California, San Diego Human Research Protections Program.

Participants for this study (*N* = 40) each had one brain scan (i.e., baseline scan) acquired before the adolescent had any significant alcohol or drug use, and one follow-up scan approximately 3 years later after half transitioned into heavy substance use, for a total of 80 scans. At baseline, inclusionary criteria included being between the ages of 12 and 17 and having minimal to no experience with substances: ≤10 total drinks in their life, never with more than 2 drinks in a week; ≤5 lifetime experiences with marijuana and none in the past three months; ≤5 lifetime cigarette uses; and no history of other intoxicant use (see Table 2). Youth with any indication of a history of a DSM-IV (American Psychiatric Association, 1994) Axis I disorder, determined by the NIMH Diagnostic Interview Schedule for Children – version 4.0 (Shaffer et al., 2000) were excluded, as were youth who had any indicator of prenatal substance exposure, any history of traumatic brain injury, loss of consciousness (>2 min), learning disorder, migraine, neurological problem, serious medical condition, or were taking a medication that could alter brain functioning or brain blood flow. After screening, approximately 12% remained eligible (see Table 1). Participants in the larger study completed substance use interviews every 3 months, and those who started heavy drinking were selected for a comprehensive annual follow-up with neuroimaging, and matched to a non-using control subject on baseline and follow-up age and pubertal development level, gender, race, family history of alcohol use disorders, and socioeconomic status. At follow-up, 20 were defined as heavy drinkers; 20 continuous non-drinkers were selected to match the characteristics of the heavy drinkers (see revised Cahalan classification used in Squeglia et al., 2012a, Squeglia et al., 2009).

**Table 1:** Demographic characteristics at baseline and follow-up.

	Controls (*n* = 20)		Heavy drinkers (*n* = 20)	
	*M*	SD	*M*	SD
Gender (% female)	35%		40%	
Race (% Caucasian)	70%		70%	
Family history of alcoholism (%)	25%		35%	
**Baseline**				
Age (range: 12–17)	14.94	1.50	15.07	1.25
Hollingshead index of social position score	26.20	17.51	19.35	11.41
Parent annual salary ($)	101.40	68.34	105.25	45.53
WASI Vocabulary *T*-score	53.00	10.01	59.91	5.28
Grade point average	3.70	0.49	3.56	0.45
Females’ Pubertal Development Scale total	4.00	0.63	4.67	0.50
Males’ Pubertal Development Scale total	3.38	0.96	3.55	0.52
Beck Depression Inventory total	1.65	2.01	2.15	2.72
Spielberger State Anxiety total	26.80	5.24	26.95	5.66
CBCL/ASR internalizing *T*-score	44.94	9.44	45.74	10.73
CBCL/ASR externalizing *T*-score	39.06	6.85	44.00	8.42
**Follow-up**				
Age (range: 15–21)	17.15	1.55	18.03	1.96
Grade point average	3.44	0.49	3.43	0.56
Female Pubertal Development Scale total	4.43	0.53	4.67	0.52
Male Pubertal Development Scale total	4.08	0.79	4.10	0.57
Beck Depression Inventory total	1.30	1.66	2.75	4.00
Spielberger State Anxiety total	25.10	5.59	23.35	3.22
CBCL/ASR internalizing *T*-score	42.84	8.10	40.74	8.95
CBCL/ASR externalizing *T*-score	42.53	7.28	46.63	10.26

*Abbreviations*: WASI, Weschler Abbreviated Scale of Intelligence; CBCL, Child Behavior Checklist; ASR, Adult Self Report.

*Note*: Hollingshead scores: higher scores represent lower socioeconomic status; annual salary based on $100 K US dollars; grade point average on a 0–4.0 scale, with higher scores corresponding to better grades; pubertal development scores range from 1 to 5, with higher numbers corresponding to more mature developmental levels. Beck Depression Inventory and Spielberger State Anxiety scores: higher scores represent more depressive or anxiety symptoms.

Ethnicity was: 70% Caucasian, 8% Latino/a, 4% Asian, and 18% multiracial (no significant group differences).

*Note*: There were no significant group differences on any demographic variable.

### 2.2 Measures

#### 2.2.1 Substance use measures

The Customary Drinking and Drug Use Record (Brown et al., 1998) obtained self-report of quantity and frequency of lifetime and recent (past 3-month) alcohol, tobacco, and other drug use (i.e., amphetamines, barbiturates, hallucinogens, cocaine, inhalants, opiates, spice, benzodiazepines, ecstasy, ketamine, gamma hydroxybutyrate, and other misused prescription medications), withdrawal/hangover symptoms, and endorsement of abuse and dependence criteria. The Timeline Followback (Sobell and Sobell, 1992) assessed substance use for the 30 days prior to the scan session, and a parent report of youth substance use was collected as collateral evidence. Breathalyzer and urine toxicology screens confirmed self-report data.

#### 2.2.2 Family background

The Family History Assessment Module (Rice et al., 1995) ascertained familial density of alcohol (AUD) and other substance use disorders (SUD) by adding 0.5 for each biological parent and 0.25 per biological grandparent (Zucker et al., 1994) endorsed by either youth or parent as having AUD or SUD. Family history data were collected from one parent, plus the other parent or (in <7% of cases) another close relative. Socioeconomic background (i.e., educational attainment, occupation, and salary of each parent) was obtained from parents (Hollingshead, 1965).

#### 2.2.3 Development

The Pubertal Development Scale (Petersen et al., 1988) provided a reliable and valid 5-item self-report measure of pubertal maturation, correlating well with physician ratings and Tanner Sexual Maturation Scale self-ratings (Miller et al., 1988). Scores ranged from 1 (prepubertal) to 5 (postpubertal). Participants in this sample were, on average, mid- to late-pubertal at baseline, and late- to post-pubertal at follow-up.

#### 2.2.4 Psychopathology and mood

The Child Behavior Checklist (CBCL; Achenbach and Rescorla, 2001) was completed by parents for youth under age 18, and Adult Self Report (Achenbach and Rescorla, 2001) was completed by youth over age 18. These measures provided level of adolescent psychopathological syndromes (e.g., internalizing and externalizing behaviors).

#### 2.2.5 Cognition

To measure executive functioning (e.g., inhibition, set-shifting, and complex problem solving), the Delis–Kaplan Executive Function System (D-KEFS; Delis et al., 2001) Trails Letter-Number Switching (Condition 4) time to complete (seconds), Color Word Interference Inhibition and Inhibition/Switching (Conditions 3 and 4), and Towers Total Achievement raw scores were acquired.

### 2.3 Image acquisition

All imaging data (baseline and follow-up) were collected from the same 3-Tesla CXK4 short bore Excite-2 MR system (General Electric, Milwaukee, WI) with an 8-channel phase-array head coil at the UCSD Keck FMRI Center. Eight high bandwidth receivers for ultra-short TR times reduced signal distortions and signal dropout. Participants were placed comfortably on the scanner table and the head was stabilized within the head coil using foam cushions (NoMoCo Pillow, La Jolla, CA). Scan sessions involved a 10-s scout scan to assure good head placement and slice selection covering the whole brain, followed by a sagittally-acquired high-resolution *3d* T1-weighted anatomical MRI that lasted 7 min and 26 s (FOV 24 cm, 256 × 256 × 192 matrix, 0.94 mm × 0.94 mm × 1 mm voxels, 176 slices, TR = 20 ms, TE = 4.8 ms; flip angle 12°). Total scan time was ∼60 min.

### 2.4 Follow-up procedures

Participants were assessed using rigorous follow-up procedures (Kleschinsky et al., 2009, Twitchell et al., 1992), with an overall follow-up rate of 99% through Year 6. Specifically, every three months after the baseline interview and imaging were complete, participants were interviewed to assess current substance use and psychiatric functioning. Those who met criteria for heavy drinking (see Squeglia et al., 2009 for classification) were invited to return and complete annual full in-person assessments (see Section 2.2), including neuroimaging. Each participant that endorsed heavy drinking was matched to a demographically similar participant who continued to endorse no substance use throughout the follow-up (i.e., continuous non-drinkers) for comparison. Moderate drinkers were excluded from analysis in this paper.

### 2.5 Image processing

Images were first reviewed for quality; images with excessive subject motion or artifact were excluded from analysis (*n* = 2, not described in this paper), leaving the 40 total subjects used in analysis. Next, images were automatically corrected for spatial distortion due to gradient nonlinearity (Jovicich et al., 2006) and B1 field inhomogeneity (Sled et al., 1998), using an in-house processing stream. Volumetric segmentation (Dale et al., 1999, Fischl et al., 2002, Fischl et al., 1999, Fischl et al., 2004) based on the publicly available FreeSurfer software package were used to generate volumetric measures (also known as automated segmentation or ASEG) for a total of 49 regions of interest (ROIs) per hemisphere, of which a list can be found in (Fischl et al., 2002) and at the FreeSurfer website, http://surfer.nmr.mgh.harvard.edu. Cortical parcellations were obtained using an in-house software package with regions derived from the Desikan atlas (Desikan et al., 2006). Qualitative review was employed to ensure that there was no technical failure of the application.

FreeSurfer 4.5.0 was used and required ∼24-h computational time for image construction, using a dual quad core Intel(R) Xeon(R) CPU E5420 with a processing speed of 2.50 GHz and 16 GB RAM. Use of several CPUs allowed processing of multiple subjects’ scans to occur in parallel. Subtle longitudinal morphometric changes in brain structure were measured by using a method developed at UCSD's Multimodal Imaging Laboratory, called “quantitative anatomic regional change analysis,” or QUARC (Holland et al., 2011, Holland et al., 2012). In the QUARC procedure, each subject's follow-up image is registered to the baseline image using a 12-parameter affine registration and then intensity normalized to the baseline image by an iterative procedure. A deformation field is then calculated from the nonlinear registration (Holland et al., 2011), and used to align the images at the subvoxel level, resulting in a one-to-one correspondence between each vertex on the baseline and follow-up images. Subcortical segmentation and cortical parcellation labels from the baseline image were used to extract an average volume change for each region of interest. A visual quality control in the volume change field was performed by a trained technician and supervised by an image analysis expert (A.M.D., 20 years experience).

### 2.6 Statistical analyses

#### 2.6.1 Demographic and substance use differences at baseline and follow-up

*T*-tests were used to compare group differences in substance use and demographic variables at baseline and follow-up.

#### 2.6.2 Baseline neuroanatomical differences

ROI values were generated from FreeSurfer output and exported to SPSS. Multivariate analysis of variance (MANOVA) was used to evaluate differences between continuous controls (*n* = 20) and heavy drinking transitioners (*n* = 20) in the 49 FreeSurfer ROI volumes per hemisphere at baseline, before any of the youth had initiated alcohol use.

#### 2.6.3 Longitudinal volume change

QUARC analysis provided percent volume change in each FreeSurfer ROI for each subject, similar to FreeSurfer output. These values were exported to SPSS for statistical analysis. MANOVA was used to evaluate group differences in volume change for each of the 49 ROIs per hemisphere. For this initial exploratory longitudinal examination, α was set at .05.

#### 2.6.4 Relationship between volume change and substance use

Pearson *r* correlations examined the relationship between changes in brain volumes and indices of drinking behavior for heavy drinkers (*n* = 20) over the follow-up period (see follow-up drinking variables in Table 2).

**Table 2:** Substance use characteristics at baseline (ages 12–17) and follow-up (ages 15–21).

	Controls (*n* = 20)		Heavy drinkers (*n* = 20)	
	*M*	SD	*M*	SD
**Baseline**				
Lifetime drinks	1.05	4.47	1.20	2.71
Lifetime cannabis use occasions	0.25	1.12	0.05	0.22
Lifetime other drug use occasions	0.00	0.00	0.00	0.00
**Follow-up**				
Lifetime alcohol use occasions**	1.75	6.46	67.40	55.25
Peak drinks on an occasion, past year**	0.50	1.24	10.90	5.16
Average drinks per occasion, past month**	0.33	0.97	4.73	2.32
Days since last alcohol use**	119.25	89.36	37.10	66.65
Cigarettes per day, past month	0.00	0.00	0.20	0.62
Lifetime cannabis use occasions**	0.35	1.14	26.55	65.70
Cannabis use days, past month**	0.00	0.00	2.40	6.10
Used cannabis >5 times (%)**	0%		20%	
Lifetime other drug use occasions	0.00	0.00	0.70	2.45

*Note*: Other drugs included: amphetamines, barbiturates, hallucinogens, cocaine, inhalants, opiates, spice, benzodiazepines, ecstasy, ketamine, gamma hydroxybutyrate, and other misused prescription medications.

** Continuous controls ≠ heavy drinkers, *p* < .01.

#### 2.6.5 Relationship between baseline volume and cognitive functioning

Pearson *r* correlations examined the relationship between baseline volume and executive functioning performance for all participants (*N* = 40) on cognitive variables of interest (see Section 2). For this exploratory examination, α was set at .01.

## 3 Results

### 3.1 Demographic and substance use differences at baseline and follow-up

At baseline, continuous controls and heavy drinkers were well-matched, as there were no significant differences in demographics (see Table 1) or substance use between groups (i.e., both had no to minimal substance use). As expected, substance use was significantly different between groups at follow-up (*p* < .001) (see Table 2).

### 3.2 Baseline neuroanatomical differences

**Fig. 1:**
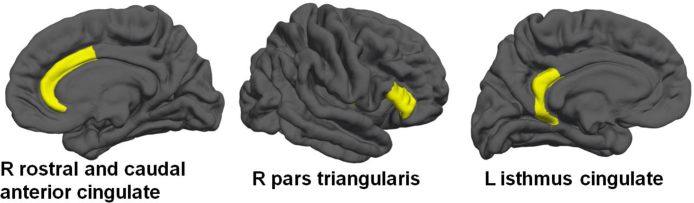
Cortical brain regions (in yellow) showing significantly less volume at baseline (ages 12–17) in youth who would initiate heavy drinking over the three year follow-up, when compared to youth who would remain continuous controls. (For interpretation of the references to color in this figure legend, the reader is referred to the web version of the article.)

While there were no overall significant baseline volume differences between heavy drinkers and controls in the full model including all brain regions [*F*(1, 38) = 0.83, *p* = .72; Wilk's *Λ* = 0.03, partial *η*^2^ = .97], there were significant volume differences between groups in five brain regions (see Table 3). At baseline (i.e., when all subjects were still non-drinkers), subjects who transitioned into heavy drinking by the three year follow-up had smaller brain volumes in the right rostral anterior cingulate, right caudal anterior cingulate, right pars triangularis, and left isthmus cingulate, and had less right cerebellar white matter (*p* < .05), as compared to youth who remained continuous controls over the follow-up period (see Table 3 and Fig. 1).

**Table 3:** Volumes (mm^3^) at baseline (ages 12–17; prior to any substance use) for those who would remain non-users versus those who would transition into drinking.

Region	Continuous controls (*n* = 20) Mean (SD)		Heavy drinking transitioners (*n* = 20) Mean (SD)		*p*	*η*^2^
Right rostral anterior cingulate	2221.46	313.67	1921.25	360.27	0.008	0.16
Right caudal anterior cingulate	2213.76	442.25	1906.30	406.20	0.028	0.13
Right pars triangularis	4717.22	1123.16	3982.82	811.87	0.024	0.13
Left isthmus cingulate	3243.71	607.25	2847.20	482.49	0.028	0.11
Right cerebellar white matter	15,880.30	1801.92	14,747.20	1721.96	0.049	0.09

### 3.3 Longitudinal volume change

**Fig. 2:**
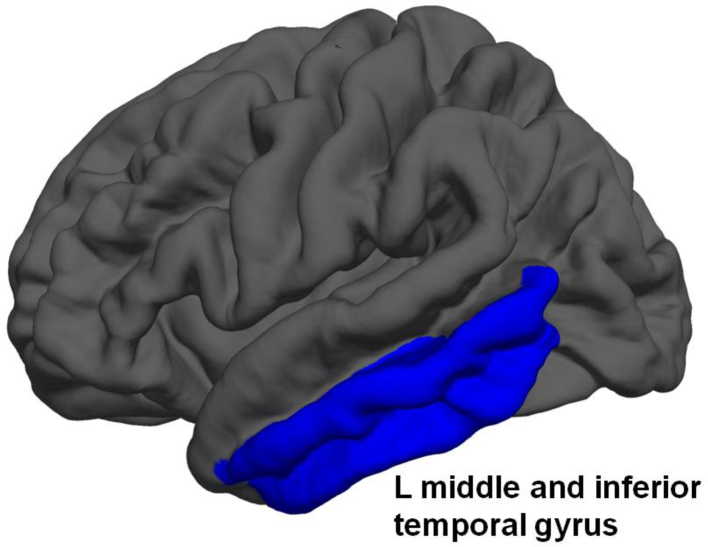
Cortical regions in blue (i.e., left middle and inferior temporal gyrus) showing significant difference in change over time (baseline ages 12–17 to follow-up ages 15–21) between adolescents who remained non-users, and those who initiated drinking over the follow-up. (For interpretation of the references to color in this figure legend, the reader is referred to the web version of the article.)

While the overall model including all brain regions was not significant [*F* (1, 38) = 3.62, *p* = .40; Wilk's *Λ* = 0.01, partial *η*^2^ = .99], there were significant changes in brain volume found across time points between groups in five areas. Youth who initiated heavy drinking over the follow-up showed significantly more reduction in volumes of the left ventral diencephalon, left inferior and middle temporal gyrus, left caudate, and brain stem (*p* < .05; see Table 4 and Fig. 2).

**Table 4:** Brain regions showing significant percent change in volume from baseline (ages 12–17) to follow-up (ages 15–21), between adolescents who remained non-users and those who initiated drinking. Most youth showed volume reductions (i.e., negative percent change over time), consistent with normal cortical thinning and synaptic pruning during adolescence, but those who started drinking heavily showed accelerated volume reductions.

Region	Continuous controls (*n* = 20) Mean (SD)		Heavy drinking transitioners (*n* = 20) Mean (SD)		*p*	*η*^2^
Left ventral diencephalon	0.002	0.011	−0.006	0.007	0.011	0.16
Left inferior temporal gyrus	−0.013	0.017	−0.026	0.014	0.012	0.16
Left middle temporal gyrus	−0.012	0.022	−0.027	0.022	0.031	0.12
Left caudate	−0.016	0.014	−0.026	0.013	0.026	0.12
Brain stem	0.008	0.013	−0.001	0.012	0.030	0.12

### 3.4 Relationship between volume change and substance use

**Fig. 3:**
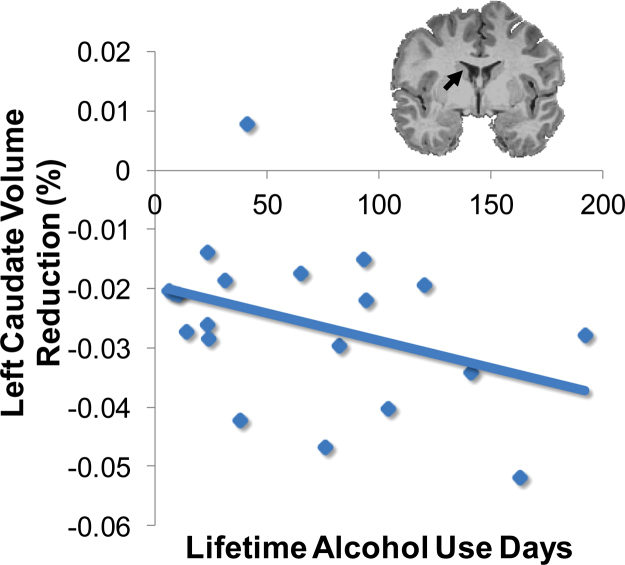
For heavy drinkers (*n* = 20), more lifetime alcohol use occasions was linked to greater volume reduction of the left caudate (*r* = −.38, *p* < .05).

Substance use during the follow-up interval significantly correlated with volume changes (i.e., in brain regions listed in Table 4) for drinkers (*n* = 20). More lifetime alcohol use occasions were linked to greater volume reduction of the left caudate (*r* = −.38, *p* < .05; see Fig. 3) and brain stem (*r* = −.38, *p* < .05). In contrast, lifetime cannabis and other drug use showed a positive relationship with brain volume; more lifetime cannabis and other drug use was linked to increasing volumes of the left caudate (*r* = .50, and .63, respectively; *p*s < .05), although these were driven by the relatively small number of subjects who had used other drugs (*n* = 4).

### 3.5 Relationship between baseline volume and cognitive functioning

At baseline, smaller right rostral anterior cingulate volume was related to slower total times on the D-KEFS Trails Letter-Number Switching Task (*r* = .37, *p* < .01).

## 4 Discussion

The goal of the present study was to use a recently developed longitudinal MRI paradigm (QUARC) (Holland et al., 2011, Holland et al., 2012) to investigate brain volume differences pre- and post-substance use initiation to disentangle normal adolescent cortical thinning from alcohol-related brain changes. Cortical pruning is a key component of adolescent neural development (Giedd, 2004, Jernigan and Gamst, 2005, Ostby et al., 2009); however, the heavy drinking group showed exaggerated volume reductions in these areas when compared to controls, consistent with findings from adolescent (Luciana et al., 2013) and adult populations (Fortier et al., 2011, Pfefferbaum et al., 1997). Overall, adolescent drinkers showed greater volume reductions than demographically matched controls over the ∼3 year follow-up period in the left ventral diencephalon, left inferior and middle temporal gyrus, left caudate, and brain stem. These volumetric changes were negatively correlated with lifetime alcohol use and peak number of drinks on an occasion in the past year, suggesting a dose-dependent effect of substance use on cortical thinning. These findings suggest a possible effect of alcohol on neural pruning, in a way that amplifies cortical volume reductions during adolescence. These results parallel previous longitudinal functional MRI findings showing increasing brain activation over time in adolescents who initiate heavy drinking (Squeglia et al., 2012a, Wetherill et al., 2013b). These observed alcohol-related cortical reductions may help explain why youth required greater brain activation to perform at the same level as abstinent youth (i.e., hyperactivation of regions to compensate for volume reductions).

The regions showing alcohol-related volume reductions included subcortical structures (e.g., diencephalon and caudate), which are important for sensory integration, motor control, feedback processing, and habit learning, as well as inferior and middle temporal cortical structures important in visual object recognition and language comprehension. Previous findings suggest alcohol use interferes with language (Moss et al., 1994) and visuospatial (Tapert et al., 2002) abilities during adolescence, which are consistent with the brain regions found in this study; continued volume reductions related to sustained drinking during adulthood might also relate to motor issues and spatial impairments found in adult alcoholics (Sullivan et al., 2010). Volume reductions in the caudate parallel findings from adult alcoholics (Sullivan et al., 2005), while reduced medial temporal volumes parallel previous results seen in adolescent heavy drinkers (De Bellis et al., 2000, Nagel et al., 2005).

While the cause of the accelerated cortical thinning is unclear, alcohol-induced dysregulated developmental timing may be responsible for the observed effects (Goodlett et al., 2005). NMDA receptor functioning could help explain accelerated thinning in heavy drinkers, as NMDA is vital for strengthening synapses and contributing to the loss of less important connections throughout development (Stoneham et al., 2010). Thus, it is possible that repeated alcohol exposure during adolescence may interfere with normal NMDA-mediated synaptic pruning.

Baseline group differences were found in several frontal cortical volumes. Specifically, youth who initiated heavy drinking over the follow-up showed smaller cortical volume in three frontal regions, as well as less cerebellar white matter volume, when compared to youth who remained substance-naïve over the follow-up. At baseline, smaller right rostral anterior cingulate volume was related to poorer performance on a test of executive functioning (e.g., set-shifting, cognitive flexibility). These findings suggest heavy drinking youth have subtle brain abnormalities that exist prior to the onset of drinking. These findings are highly consistent with other recent functional MRI findings which found pre-existing lower frontal brain activation in teens who later initiated heavy drinking when compared to continuous controls over a three year follow-up (Norman et al., 2011, Squeglia et al., 2012a, Wetherill et al., 2013b). The current findings (i.e., smaller volumes in frontal regions, as well as reduced cerebellar white matter volume) might help explain previous findings where heavy drinking transitioners showed less brain activation in frontal regions before they initiated alcohol use. Furthermore, the frontal regions found in this study (i.e., rostral and caudal anterior cingulate, pars triangularis) are important brain regions for executive control, including inhibitory functioning, attention, impulsivity, and self-regulation (Fjell et al., 2012, Goldberg, 2001). Poorer inhibitory functioning in substance-naïve youth has been found to be predictive of future substance use (Squeglia et al., 2014), and structural brain differences could help explain these behavioral findings.

Limitations should be noted. Although overall groups were very well matched, follow-up lifetime cannabis use days (average: controls = <1; heavy drinkers = 27) significantly differed between groups. Cannabis use was related to increasing volume over time, possibly countering the volume reductions related to alcohol use. There is research that suggests cannabis may act as a protective factor for white matter integrity in binge drinking (Jacobus et al., 2009); therefore, volume reductions may have been even more pronounced if we had a completely non-cannabis using comparison group. There are also statistical limitations to be considered in this preliminary study. Findings did not survive Bonferroni or false discovery rate correction; however, the processing technique utilized is highly sensitive to morphometric brain changes, as each subject's follow-up image was registered to the baseline image. Furthermore, a typical cubic millimeter of gray matter in an adult contains 35–70 million neurons and almost twice as many glial cells (Lenroot and Giedd, 2006, Pakkenberg and Gundersen, 1997), as well as over 500 billion synapses (Scheff et al., 2001), so even slight differences in cortical thickness could be associated with significant divergence from typical synaptic pruning and gray matter loss across adolescent development. Previous findings suggest that female heavy drinkers may be more vulnerable to aberrant cortical thinning than male drinkers (Squeglia et al., 2011, Squeglia et al., 2012b). Unfortunately, our sample size (six females per group) did not allow sufficient power to detect gender effects. The parent study is ongoing and will offer larger sample sizes with more equal gender distributions, which will allow us to more fully address the moderating role of gender on the relationship between drinking and cortical thinning during adolescence. Additionally, the sample is comprised of healthy, high functioning adolescents, so findings may not generalize to clinical or lower functioning samples. The observed pattern of results may be more pronounced in those with higher levels of drinking (e.g., adolescents with AUD). Despite these limitations, these findings have important clinical and public health implications, particularly given the participants’ limited, sub-diagnostic alcohol use, limited other substance use, and absence of psychopathology. Further work with larger populations is needed to increase statistical power to observe moderating effects of variables of interest (e.g., gender) and help advance the understanding of the relationship between alcohol exposure and brain morphometry, and subsequent cognitive functioning.

## Conflict of interest

There are no known conflicts of interest associated with this publication.
